# Influence of Nano Silica Particles on Durability of Flax Fabric Reinforced Geopolymer Composites

**DOI:** 10.3390/ma12091459

**Published:** 2019-05-06

**Authors:** Hasan Assaedi, Thamer Alomayri, Faiz Shaikh, It-Meng Low

**Affiliations:** 1 Department of Physics, University College in AlJumum, Umm Al-Qura University, P.O. Box 715, Makkah 21955, Saudi Arabia; hsassaedi@uqu.edu.sa; 2 Department of Physics, Faculty of Applied Science, Umm Al-Qura University, P.O. Box 715, Makkah 21955, Saudi Arabia; tsomayri@uqu.edu.sa; 3 Department of Civil Engineering, Curtin University, GPO Box U1987, Perth 6845, Australia; s.ahmed@curtin.edu.au; 4 Department of Physics and Astronomy, Curtin University, GPO Box U1987, Perth 6845, Australia

**Keywords:** geopolymer, nano silica, mechanical properties, flax fibres, durability

## Abstract

The durability of natural fibres as reinforcement in geopolymer composites continues to be a matter of concern due to the alkalinity of activators of geopolymer matrices. The alkaline environment is the main reason for natural fibres degradation in cementitious matrices. This paper presents the influence of nano silica (NS) on the durability and mechanical performance of geopolymer composites that are reinforced with flax fabric (FF). The durability investigations were conducted after the storage of samples at ambient temperature for 32 weeks. The study revealed that the addition of nano silica has a positive influence on the physical and mechanical properties of these composites. The presence of NS accelerated the geopolymeric reaction and lowered the alkalinity of the system, thus reducing the degradation of flax fibres.

## 1. Introduction

Geopolymer is formed by activating aluminosilicate based-materials such as fly ash, volcanic ash and meta-kaolin with an alkaline solution, thereby creating amorphous structures of AlO_4_ and SiO_4_ molecules that get connected through oxygen atoms [1]. Duxson et al. [2] described a model for the geopolymerisation process in a few steps. This process involves the dissolution of aluminosilicate by alkaline solution and the concomitant release of aluminate along with silicate species. Subsequently, a mixture of the dissolved species with the aluminosilicate material is formed in the alkaline environment, which leads to a growth of a gel as the oligomers in the aqueous stage develop networks through the polymerisation process. Even as the chemical reaction progresses, water is released and consumed in the dissolution process. Reformation of the initial gel to improved gel arises by the release of additional water. Lastly, polymerisation through a condensation and hardening stage occurs. Even though the procedure is executed in sequential steps, these phases could occur simultaneously and heterogeneously.

Despite their potential and attractive properties, geopolymers continue to exhibit limitation in terms of brittle failure and low tensile and flexural strengths [3,4]. This identified limitation can be overcome by using natural fibres to fabricate natural fibres reinforced geopolymer composites. The use of natural fibres in composites is advantageous due to their flexibility and lower density and higher specific modulus [5,6]. Several natural fibres have been used successfully used in geopolymer matrices. Properties of geopolymer composites, including mechanical performance and fracture toughness, have been improved through the use of cotton fibres and fabrics as reinforcement [7,8]. Positive effects of wool and flax fibres were evident after they were incorporated into geopolymer matrices, which caused the geopolymer composites to experience a significant improvement in their mechanical properties [9,10]. Another study focused on reinforcing geopolymer composites using woven flax fabric, and testing was undertaken on mechanical properties, including compressive strength, fracture toughness, flexural modulus, hardness, and flexural strength. The findings revealed an improvement in each of the stated mechanical properties when the contents of flax fibres were increased [11].

However, it must be noted that concerns have arisen regarding use of alkali-based natural fibres in matrices. The concern is in relation to the long-term durability of composites that have been reinforced using natural fibres. It is possible for natural fibre degradation to occur if the concentration of alkaline solution used is too high, which can adversely affect the physical, chemical and mechanical properties of the fibres [12,13,14]. A study conducted by Gram [15] focused on degradation of natural fibre in the context of alkaline environment and stated that degradation occurs when the hemicelluloses, as well as textile, decompose, thus causing natural fibres to split into microfibrils. That effect has been witnessed in jute fibre reinforced cement composite where splitting and fibrillation of natural fibres occurred, causing a drop in the tensile strength by 76% [16]. Nano-particles can play a central role in reducing the degradation of natural fibres. The effect of nano-clay on durability of cement composites reinforced with flax fibres has been investigated by Aly and co-workers [13]. They observed that the composites containing 2.5 wt% nano-clay particles exhibited lower deterioration in the flexural performance as compared to the control samples after 50 wet and dry cycles. That, in turn, was ascribed to the impact of nano-clay in reducing the deterioration of the natural fibre and as a result, improving the durability of the composite. In our previous study, nano-clay particles were added to flax fibre reinforced geopolymer composite in order to investigate their influence on medium-term and long-term durability. It was shown that all samples experienced different levels of deterioration and reduction in their strength after being exposed to wet and dry cycled for 32 weeks. However, the reduction in the flexural strength of composites containing nano-clay was found to be less than that of control composite [17].

Nano silica is one of the most widely researched nano materials for various applications in concrete and geopolymer. In this study, nano silica (NS) is used to modify the geopolymer matrices and improve the durability of flax fibres within the geopolymer composites. The primary objective of this work is to investigate the influence of various NS contents on the mechanical properties and durability of flax fibre reinforced geopolymer composites. The durability of specimens was studied in terms of the flexural strength tested at 4 and 32 weeks. Moreover, the microstructure of composites was assessed by scanning electron microscopy (SEM), X-ray diffraction (XRD), and Fourier transforms infrared spectroscopy (FTIR).

## 2. Materials and Methods

### 2.1. Materials and Sample Preparation

The fly ash used in this study was obtained from Eraring power station of Lake Macquarie, New South Wales (NSW) in Australia and satisfied ASTM class F fly ash classification. Commercial grade D sodium silicate solution was used whose chemical composition was 55.9% H_2_O, 14.7% Na_2_O, and 29.4% SiO_2_ by mass. The sodium hydroxide was 98% pure and then mixed with potable water in order to prepare 8 molar sodium hydroxide solution. Subsequently, alkaline solution was prepared by combining sodium silicate solution with 8M sodium hydroxide solution at a ratio of 2.5:1 (by weight). NS particles were obtained from Nanostructured and Amorphous Materials Inc. (Katy, TX, USA). The particles had an average diameter in the range of 18–25 nm. 

**Figure 1:**
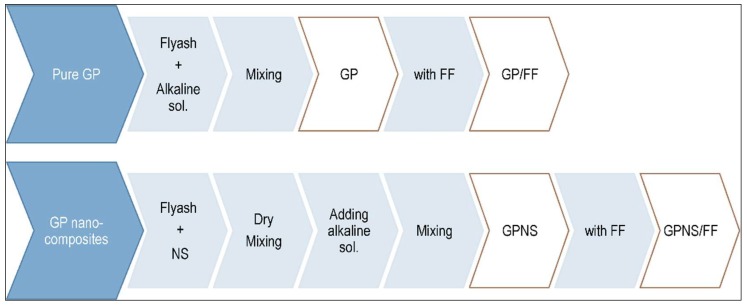
Diagram presenting the procedures of producing the samples.

Figure 1 provides a summary of the process followed in preparing all samples. In order to prepare the pure geopolymer and geopolymer nanocomposites, nano silica was added to the fly ash at 0.0%, 0.5%, 1.0%, 2.0%, and 3.0% by weight. They were placed in a covered mixer to dry-mix at a low speed for five minutes. The dry-mixing method was chosen due to its ability to ensure better dispersion of the nanoparticles with fly ash powder. They were then left for an additional 10 min to mix at high speed until the homogeneity of dry mix was obtained. The next step entailed the addition of the alkaline solution to the dry mix and mixed initially at low speed, awaiting the mixture to achieve homogeneity and then for another 10 min at high speed. The resulting mixture was poured in to wooden molds which were subsequently vibrated for two minutes on a vibrating table before covering them with a plastic sheet. These molds were then placed in oven for 24 h at 80 °C, where geopolymer reaction occurred. The geopolymer paste without nano silica particles was considered as the control sample. 

Fabrication of the flax fabric (FF) reinforced composites and the composites containing nano silica (hereafter called nanocomposites) requires the preparation of similar mixes. Four samples of geopolymers were reinforced using ten layers of flax fabric. This process required the paste being spread into a greased wooden mold followed by the first layer of flax fabric. Thereafter, a roller was used to completely saturate the fabric with the paste. The process was repeated for ten layers. In order to ensure the removal of entrapped air inside the samples, a 20 kg weight was placed on top of them. A plastic sheet was then used to cover each of samples, after which they were cured (at 80 °C) for 24 h in an oven before de-molding. The resulting samples were then dried for 28 days under ambient conditions. The geopolymer matrix that was reinforced using flax fabric without nano silica is considered as the control sample. 

The samples were subsequently categorised in two series. The samples under the first series was cured for four weeks under ambient conditions. The second series was stored under similar conditions, albeit for 32 weeks. Table 1 illustrates mix proportions of the samples.

### 2.2. Characterisation

Identical pieces have been selected and cut from the samples prepared. They were then crushed before being grounded to fine powder. A D8 Advance Diffractometer (Bruker, Germany) was used to measure the samples of powder through use of copper radiation and a position sensitive detector (the LynxEye). At 0.5°/min (in scanning rate), scanning of the diffractometer was done from 7.5° to 60°. Cu $k_{\alpha }$ lines (k = 1.5406 Å) were utilised in order to get XRD patterns. Additionally, MAUD V2.44 software was used to perform QXDA (Quantitative X-ray Diffraction Analysis) with Rietveld refinement. The compound selected to represent an internal standard was a fluorite (CaF_2_) [18]. To prepare the QXDA samples, geopolymer paste (3.0g in dry weight) was mixed with fluorite (0.33g in weight). Parameters that were defined on the basis of Rietveld were subsequently used to calculate the weight percentage of the individual crystalline phases (*W_Cr_*) through Equation (1) [19] shown below:

(1) $$W_{Cr}=\left[W_{std}\frac{S_{Cr}{\left(ZMV\right)}_{Cr}}{S_{std}{\left(ZMV\right)}_{std}}\right]\times \left[\frac{1}{1-W_{std}}\right]$$

*W_std_* denotes the standard weight per cent, M represents the mass of unit cells while V refers to their volume. Z signifies the number of units of the formula for every unit cell. *S_Cr_* denotes the scale factor for the crystalline phases, and *S_std_* represents the scale factor for the standard. Calculation of the amorphous weight content *W_Am_* was then performed based on the equation below [19]:

(2) $$W_{Am}=1-\sum_{i=1}^{n}W_{n}$$

where *n* denotes the number of refined crystalline phases.

The chemical compositions of Eraring fly ash were analysed using X-ray fluorescence (XRF measurements were conducted externally in Bureau Veritas, Perth, Australia). FTIR scan was conducted using a FTIR spectrometer (the Perkin Elmer Spectrum 100, Perkin Elmer, Waltham, MA, USA) between the 4000–500 cm^−1^ range and at room temperature. The samples were examined in order to determine fracture surfaces and microstructures by using SEM (the Zeiss EVO-40 from Carl Zeiss, Oberkochen, Germany). The samples of geopolymer were put in a vacuum desiccator for two days to complete out-gassing before mounting the samples on aluminium stubs, which was then followed by coating with a thin platinum layer.

### 2.3. Physical and Mechanical Properties

Porosity and bulk density were measured to define the quality of geopolymer nanocomposite. Equation (3) shown below was used to calculate the density of the samples:

(3) $$\rho =\frac{m_{d}}{V}$$

where *ρ* represents the density of samples, V and md denote the volume and the dry mass, respectively.

To calculate P_a_ (value of apparent porosity), Archimedes principle was used based on the ASTM Standard (C-20) [20]. The samples of nanocomposite and pure geopolymer were immersed into clean water and Equation (4) was then used to calculate P_a_:

(4) $$P_{a}=\frac{m_{a}-m_{d}}{m_{a}-m_{w}}\times 100$$

where *m_a_* and *m_w_* denote the mass of saturated samples in the atmosphere and water, respectively. 

The mechanical tests were performed with 0.5 mm/min rate of displacement using a LLOYD Material Testing Machine (AMETEK, Berwyn, PA, USA) that has a capacity of 50 kN. Rectangular bars (whose dimensions are 60 × 18 × 15 mm^3^) were cut from the samples that were fully cured to conduct a three-point bending test in order to evaluate flexural strength. Five samples from each group were used to evaluate the flexural strength of geopolymer composites. The machine software (NEXYGENPlus, V2.0) was used to analyse the results after they were recorded. Equation (5) was used to calculate the flexural strength (σ_F_) [21]:

(5) $$\sigma_{F}=\frac{3}{2}\frac{P_{m}S}{WD^{2}}$$

where *P*_m_ represents the maximum load, *S* signifies the sample’s span, *D* and *W* denote the width and thickness of the specimen, respectively.

## 3. Results

### 3.1. Physical Properties

**Figure 2:**
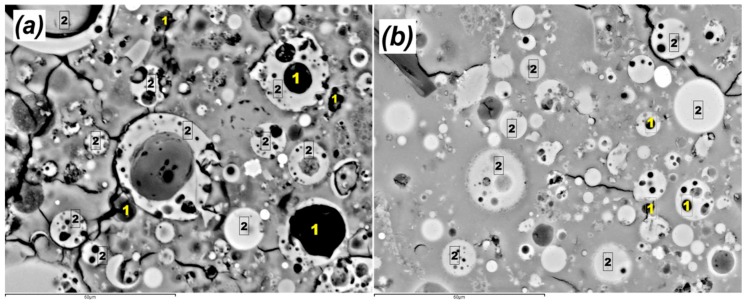
SEM images showing the microstructure of (**a**) GP and (**b**) GPNS-3.0 [25]. Numbers indicate: 1 = voids; 2 = fly ash particles.

The bulk density and porosity of pure geopolymer as well as that containing NS are shown in Table 2. Evidently, the geopolymers containing NS exhibited higher density and lower porosity as compared to the control sample. The ideal content was found as 1.0 wt% of nano silica, which creates denser matrix by about 15% and decreased the porosity by 27% as compared to the pure geopolymer. That improvement might be attributed to two reasons. First, the nanoparticles filled the pores in the matrices, which reduced the porosity of geopolymer nanocomposites. Secondly, the additional silica improved the geopolymeric reaction, thus creating further geopolymer gel and denser matrix accordingly [22]. However, further increase in NS content slightly reduced the density due to the inadequate dispersion and the agglomeration of the nano silica particles. The finding of the current study is in consonance with the observations of Supit and Shaikh [23], who found in their study that loading of 2.0 wt.% NS considerably decreased the concrete porosity. In a similar investigation, the porosity of cement paste was found to reduce due to the addition of nano-clay particles to the samples; the porosity increased with a corresponding increase in nano-clay owing to the influence of agglomeration [24]. Figure 2a,b illustrates the microstructures of pure geopolymer, and that contained 3.0 wt.% NS. Higher number of voids, un-reacted fly ash particles can be seen in Figure 2a in the case of neat geopolymer sample. However, fewer number of fly ash particles with higher amount of geopolymer gel were seen in the nanocomposite matrix (Figure 2b).

### 3.2. Characterisation and Microstructure

**Figure 3:**
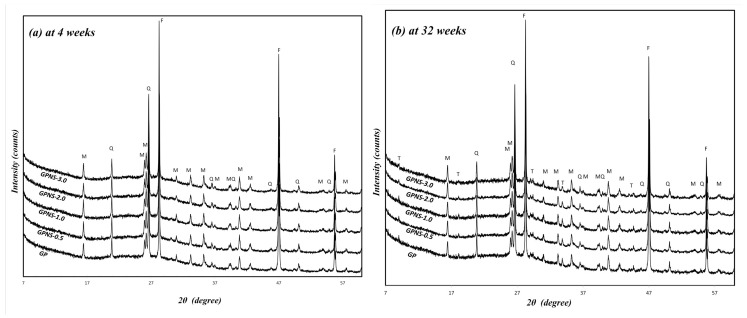
X-ray diffraction patterns of the control sample and geopolymer with various contents of nano silica: (**a**) at four weeks; (**b**) at 32 weeks. Legend: F = Fluorite, M = Mullite, Q = Quartz and T = Trona.

The chemical compounds in Eraring fly ash, as characterised by XRF, were 63.1 wt.% SiO_2_, 24.8 wt.% Al_2_O_3_, 2.58 wt.% CaO and 3.07 wt.% Fe_2_O_3_. The XRD spectra taken for these samples at both four and 32 weeks are illustrated in Figure 3a,b. Crystalline phases in these samples were identified in XRD analysis. Fluorite [CaF_2_] was the standard used for calculating the weight fraction of each crystalline phase. At the end of four weeks, all samples contained two crystalline phases, mullite [Al_2.32_Si_0.68_O_4.84_], and quartz [SiO_2_] (PDF 00-046-1045). Mullite and quartz can be observed in all specimens since they are an essential content in the Eraring fly ash. Thus, they both are not reactive in the alkaline media [26]. However, a further crystalline content called trona [Na_3_H(Co_3_)_2_ 2H_2_O] developed on the surface of the pure geopolymer and nanocomposites after 32 weeks. It may be noted that Trona is one of the soda minerals groups formed by the atmospheric reaction of carbon dioxide with the residual water and sodium hydroxide within the system [27].

**Figure 4:**
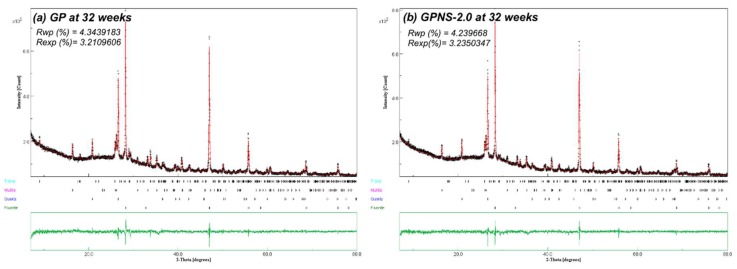
XRD Rietveld refinements for (**a**) control sample and (**b**) GPNS-1 at 32 weeks. Experimental patterns are shown in black dots, and patterns as calculated in solid red lines. The green residual line displaying the variance between the patterns as calculated and measured experimentally.

**Figure 5:**
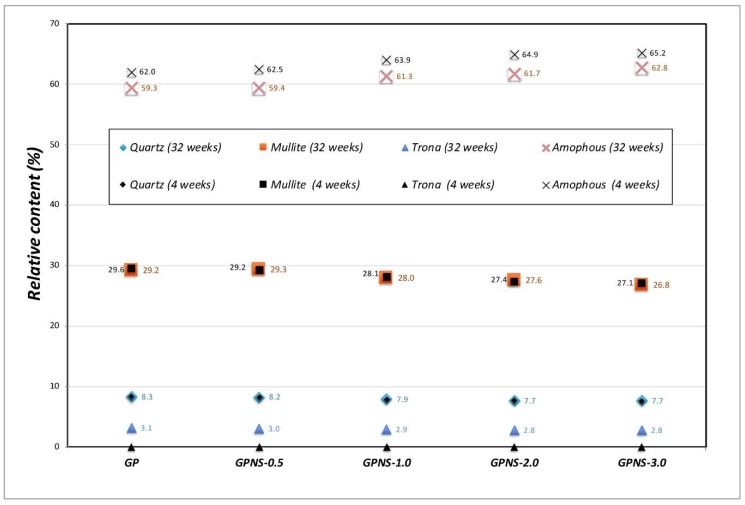
Crystalline and amorphous phases structures of the neat geopolymer and that contained various contents of nano silica at 4 and 32 weeks.

The contents of crystalline phases compositions in the specimens were quantified by Rietveld analysis refinement procedure, after which the amorphous contents were considered using Equation (2). Figure 4a,b show refined XRD patterns of the neat geopolymer, and that contained 2.0% NS at 32 weeks, respectively. Measured and calculated patterns are shown in black dots and red solid line, respectively. Intensity variances between the two patterns are shown in the residual green line at the bottom of the plot, and the vertical black bars show the allowable peak position in each crystalline phase. In general, the addition of amorphous nano silica to the geopolymer pastes led to minor changes in the crystalline and amorphous content across all specimens. Figure 5 illustrates the weighted percentage of crystalline (quartz, mullite and trona) and the amorphous content in the neat matrix and geopolymer with NS at four and 32 weeks, respectively. At the end of four weeks, the amorphous content of GPNS-3.0 increased by 3.2 wt% mainly due to the additional 3.0 wt% NS, which caused a reduction in the crystalline phase’s contents. Since nano silica is amorphous, the increase of amorphous material in the nanocomposite specimens is typically attributed to the additional NS loaded to the pastes in the capacity of a nano-filler [22,28]. The active and reacted nano silica particles could also promote the geopolymerisation reaction, thereby creating a higher amounts of amorphous geopolymeric gel within the matrices [22]. At the end of 32 weeks, the amounts of quartz and mullite remained unchanged after the ageing period. Those crystals are very stable and require high temperatures to be dissolved [29,30]. The formation of Trona crystals was primarily due to the amorphous content in geopolymers as well as the atmospheric carbon dioxide. The formation of trona can be noticed by comparing the trona and amorphous content at four and 32 weeks, respectively. After the ageing period, the amorphous amount in geopolymers reduced about 3% in all samples, which is similar to the amounts of trona grown after the ageing period. The amount of carbonation content (trona) was found to be highly contingent on the position and is chosen from each sample since trona formed on the surfaces of geopolymers. However, all XRD samples were chosen from similar parts in each composite in order to ensure they are nearly identical; for this reason, the analysis can provide a reasonable estimation of the relative crystalline and amorphous content within each sample.

**Figure 6:**
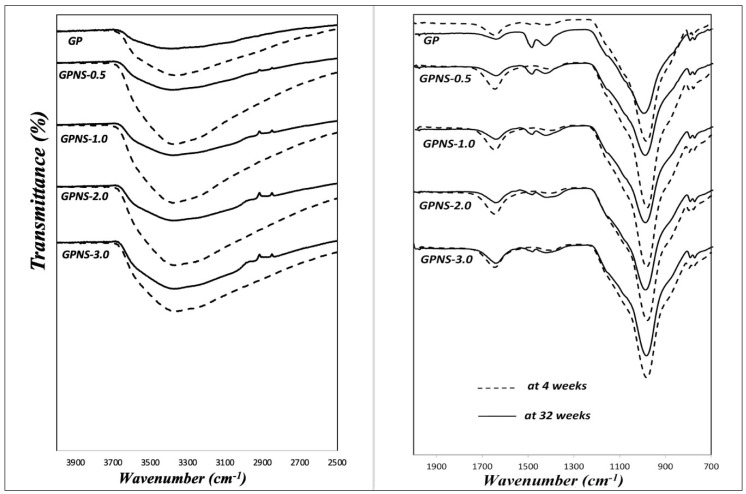
Spectra of FTIR for geopolymer sample and that containing various contents of nano silica at 4 and 32 weeks.

Figure 6 illustrates FTIR spectra of the control sample and that containing nano silica at 4 and 32 weeks, respectively. The strong peak at 1000 cm^−1^ is interpreted to an overlap of Al-O-Si and Si-O-Si asymmetric stretching vibrations. This is widely known as a distinct peak and fingerprint of geopolymers [31,32]. The widespread peak in the range of about 3400 cm^−1^ can be ascribed to the hydroxy group (OH) linkage to various centres (mainly aluminium and silicon) and free water [33,34]. The peak at 1640 cm^−1^ is an absorbent and likewise referred to the bending vibration of OH [35]. However, changes were found to occur at 34 weeks. The development of sodium carbonate can be identified based on the new two peaks at 1420 and 1480 cm^−1^, respectively. Those peaks were created by atmospheric carbonation on the surface of the matrices, which confirms the positive results of the XRD [27]. During the ageing process, the geopolymeric reaction took place at a slow rate, which consumed further hydroxy groups and formed tougher geopolymer. The water content decreased slightly to a certain level of balance throughout that period, resulting in a lower wide-ranging peak at 3400 cm^−1^. In addition, the geopolymer band at about 1000 cm^−1^ shifted slightly to higher wavenumbers due to a higher polycondensation reaction, which indicated the higher transformation of Gel 1 to Gel 2 [2,36].

### 3.3. Flexural Properties of Geopolymer Nanocomposites

**Figure 7:**
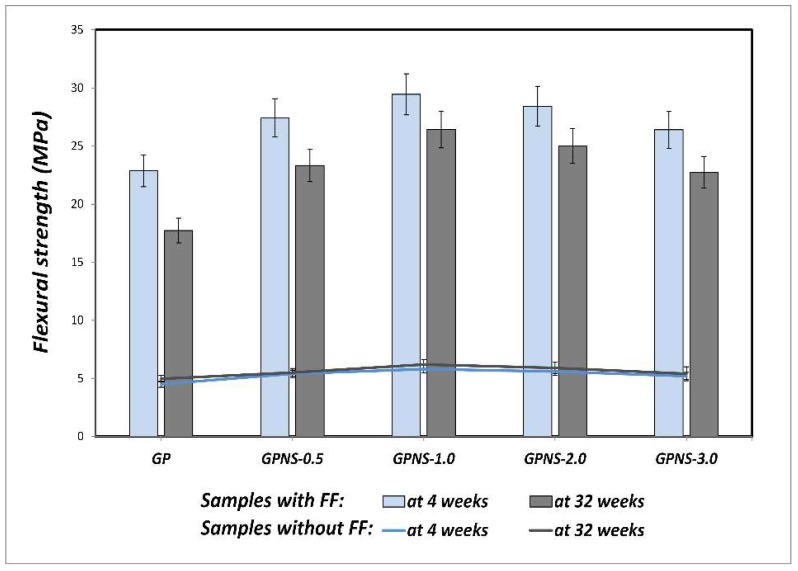
Flexural strength of all samples at 4 and 32 weeks.

Figure 7 illustrates how the flexural strength (of nanocomposites as well as pure geopolymer) is impacted by ageing. In general, a slight improvement in flexural strength was observed at all ages due to the incorporation of nano silica particles into the geopolymer composite. As compared to the control paste, the geopolymer nanocomposites in which the nano silica content was 0.5, 1.0, 2.0, and 3.0 wt.% showed an increase in flexural strength (at four weeks) by approximately 20%, 28%, 24%, and 15%, respectively. The improvement observed demonstrates that nano silica particles are effective in both enhancing geopolymeric reaction and filling the micropores in the matrices [24,26,37]. The geopolymer nanocomposite microstructure was, therefore, denser than the net geopolymer, particularly in the case of 1.0 wt.% NS, which is apparent from the observations of the flexural strength. Once again, it was seen that the the flexural strength of nanocomposites increased slightly at 32 weeks as compared to their previous results at four weeks. For example, the average flexural strength of GPNS-1 nanocomposite improved from 5.8 to 6.2 MPa (6.8% increase). This small but noticeable improvement in mechanical properties can be attributed to the reaction of both alumina and silica during the ageing period in the alkaline environment [38,39]. In a comparable investigation, Rong et al. [40] evaluated the effects of the addition of 3.0 wt.% of nano silica on the durability of concrete that contained 35 wt.% fly ash in 28 and 90 days, respectively. They reported an increase in flexural strength of almost 11% at 90 days as opposed to 28 days. Another study [41] also observed that the flexural strength of concrete, including 1.0 wt.% nano silica, increased by about 10% after 90 days, as compared to 28 days.

### 3.4. Flexural Properties of Flax Fabric Reinforced Geopolymer Nanocomposites

**Figure 8:**
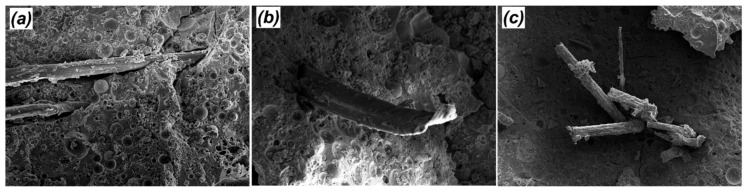
SEM images showing the fracture surfaces of flax fibres reinforced composites; (**a**) GP/flax fabric (FF), and (**b**,**c**) GPNS-1/FF [25].

The flexural strength investigations are repeatedly used to evaluate the mechanical performance of multilayered composites because they represent a simple yet efficient way of measuring the bending behaviour, providing technical results on the overall behaviour of the composites under applied force [42]. Figure 7 depicts the effect of the ageing process on the flexural strength of the samples at four and 32 weeks, respectively. The incorporation of nano silica particles into matrices resulted in a significant increase in the flexural strength of all FF reinforced nanocomposites. To illustrate, GPNS-1/FF’s flexural strength at four weeks increased from 23.0 to 30.5 MPa, which is approximately 32.4% higher than GP/FF samples. This improvement could be attributed to the enhanced density of the nanocomposites that led to improved adhesion bond between flax fibres and geopolymer nanocomposites. The fracture surfaces of GP/FF and GPNS-0.1/FF after the flexural test at four weeks are shown in Figure 8a–c, respectively. Meanwhile the fracture surface of GP/FF composite displays high porosity, voids, and particles of fly ash that are deeply rooted in the matrices. Thie increased porosity reduced the fibre-matrix bond, which, in turn, allowed fibres to debound and pull out of the geopolymer matrix, as seen in Figure 8a. However, the fabric-reinforced nanocomposites loaded with 1.0 wt.% nano silica exhibits relatively denser microstructure with less unreacted particles of fly ash. Consequently, improved adhesion between fibres and the matrix is observed, causing the fibres to be fractured (see Figure 8b,c). In both these cases, the fibres seemed uniform and did not exhibit any visible signs of degradation.

**Figure 9:**
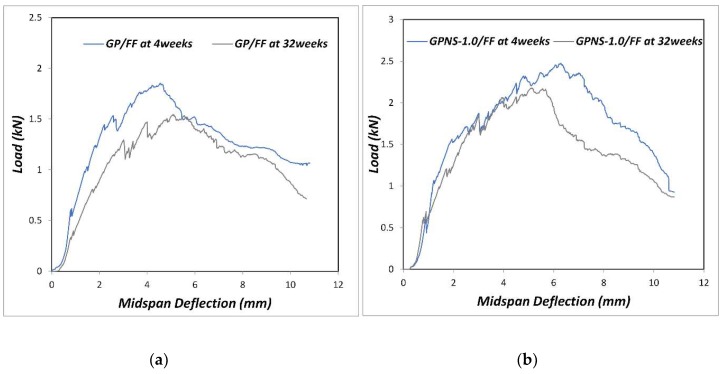
(**a**) Load—midspan deflection graph for GP/FF composite and (**b**) GPNS-1/FF nanocomposites at 4 and 32 weeks.

**Figure 10:**
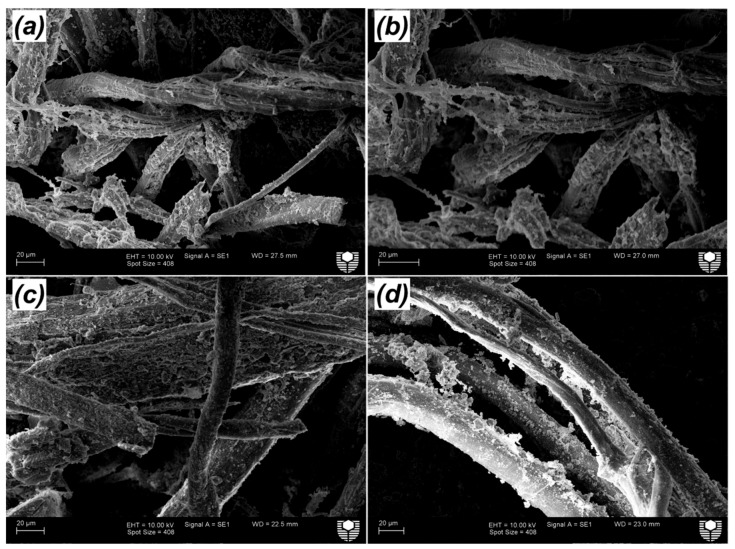
SEM images of the flax fibres at 32 weeks in: (**a**,**b**) GP/FF, (**c**) GPNS-1/FF and (**d**) GPNS-3/FF.

After the ageing period of 32 weeks, all composites showed a reduction in the flexural test. Figure 9a,b show the load-deflection behaviour of GP/FF and GPNS-1/FF composites at four and 32 weeks, respectively. The ductile behaviour can be seen with and without the nanoparticles in both composites, with a higher load capacity (31%) in the composite containing NS. The degradation process was observed to cause the bending stress as well as ductile behaviour to decline. Normally, cellulose fibres suffer different levels of deterioration after being exposed to an alkaline environment [43]. The degradation of natural fibres has been addressed by several studies and was attributed to the weakening of lignin and hemicellulose due to the attack of alkali ions, as well as the mineralisation of fibre cell walls in geopolymer pastes that led to the fibre brittleness [44,45,46]. The fibre degradation in alkali pastes eventually led to the deterioration of adhesion force that bound the fibres to the matrices, and the concomitant reduction in flexural strength of the composites. It was noticed that the flexural strength of geopolymer composite reinforced with flax fibres reduced by 22.4% of its strength at four weeks, while the flexural strength of GPNS-0.5/FF, GPNS-1/FF, GPNS-2/FF and GPNS-3/FF samples reduced by approximately 14.9%, 10.3%, 12.1% and 13.8%, respectively as compared to the corresponding results at four weeks. These results suggest that after 32 weeks (of ageing), the nanocomposites experienced less reduction in flexural strength as compared to the control sample composite. This could be because some amount of the alkaline solution was consumed by nano silica; therefore, the alkalinity of the matrix was reduced, whereas the additional silica activated the geopolymerisation, increasing the amount of geopolymer gelin in the matrix. These phenomenon noticeably improved the matrix density and in effect, the adhesion between the fibres and geopolymer matrix [26]. Figure 10a–d illustrate the changes in the surface of the fibre in GP/FF, GPNS-1.0/FF and GPNS-3.0/FF at 32 weeks. The fibres in control sample (Figure 10a,b) revealed degradation signs and separation of the small fibrils, which, in turn, influenced the flexural behaviour of the composite. On the other hand, fibres in the sample GPNS-1.0/FF and GPNS-3.0/FF did not exhibit signs of significant damage after the ageing period (Figure 10c,d). When compared this finding to our investigations in the case of incorporating nanoclay particles, it was found that there was a reduction in the flexural strength of composites of flax fibres and geopolymer by about 14% [17]. In a comparable investigation, Aly et al. [13] stated that durability was improved after the addition of nanoclay to cement mortar with waste glass. Furthermore, degradation of flax fibres was mitigated in the composites because alkalinity was reduced in the matrix. Another study found that when calcined nanoclay was added to cement composites reinforced using hemp fabric, the durability improved and the hemp fibres experienced less degradation [12]. In yet another study, when meta-kaolin was added into mortar (reinforced using sisal fibre) at 28 days and following 25 wet and dry cycles, an improvement was noted in durability [46]. The meta-kaolin composites experienced a reduction in flexural strength by 23% in comparison to the control composites at 28 days. Furthermore, it was determined that sisal fibres in cement matrices did not experience significant degradation upon the loading of 50% meta-kaolin. In the current study, the flax fibres degradation in the matrices typically reduced, while the interfacial bond between the natural fibres and geopolymer nanocomposites was enhanced.

## 4. Conclusions

The following conclusions were drawn based on the experimental work conducted and results analysis:

- Nanosilica effects on the durability of flax fabric reinforced geopolymer composites with nanosilica were established and the optimum content of nanosilica were reported.
- The physical and mechanical properties of FF-geopolymer nanocomposites subjected to long period of curing were also evaluated.
- NS was found to decrease the internal voids and densify the microstructure of geopolymer nanocomposites due to its low specific weight as compared to fly ash.
- The test results indicated a 23.01% reduction in the flexural strengths of GP/FF composites without NS after 32 weeks, while the GPNS-1/FF composites with NS experienced a 10.2% decrease in flexural strength subjected to the same period of curing.
- SEM analysis shows that the nanoparticle fills the void spaces, which result in uniform, less voids and compact geopolymer matrix.
- Based on SEM images, it was observed that the degradation of flax fabric reinforced geopolymer composites without nanosilica was higher than that with 1.0 wt.% nanosilca.
- The FTIR spectra and XRD analysis of geopolymer composites with nanoslica demonstrated that the additions of nanosilica promote the geopolymerisation reaction, thus creating a higher amount of amorphous geopolymeric gel within the matrices. This, in turn, show that nanoparticles assist in preventing the strength retrogression of geopolymer nanocomposites.

## Figures and Tables

**Table 1:** Mix proportions of samples.

Sample	Fly Ash (g)	Alkaline Solution (g)	NS (g)	Water(g)	FF (wt%)
GP	1000	750	0	50	0
GPNS-0.5	1000	750	5	50	0
GPNS-1.0	1000	750	10	50	0
GPNS-2.0	1000	750	20	50	0
GPNS-3.0	1000	750	30	50	0
GP/FF	1000	750	0	50	4.1
GPNS-0.5/FF	1000	750	5	50	4.1
GPNS-1.0/FF	1000	750	10	50	4.1
GPNS-2.0/FF	1000	750	20	50	4.1
GPNS-3.0/FF	1000	750	30	50	4.1

**Table 2:** Density and porosity values of the control sample and geopolymer containing nano-silica. Uncertainties are indicated in brackets.

Sample	Density (gm/cm^3^)	Porosity (%)
GP	1.84 (0.02)	22.20 (0.45)
GPNS-0.5	1.89 (0.02)	20.87 (1.35)
GPNS-1.0	2.10 (0.02)	16.08 (0.76)
GPNS-2.0	2.04 (0.03)	17.49 (1.84)
GPNS-3.0	1.96 (0.08)	20.33 (1.01)
